# A dichotomic approach to adaptive interaction for socially assistive robots

**DOI:** 10.1007/s11257-022-09347-6

**Published:** 2022-11-17

**Authors:** Riccardo De Benedictis, Alessandro Umbrico, Francesca Fracasso, Gabriella Cortellessa, Andrea Orlandini, Amedeo Cesta

**Affiliations:** ISTC-CNR - Institute of Cognitive Sciences and Technologies, National Research Council of Italy, Rome, Italy

**Keywords:** Personalized interaction, User modeling, Automated planning, Reactive reasoning, Socially Assistive Robots

## Abstract

Socially assistive robotics (SAR) aims at designing robots capable of guaranteeing social interaction to human users in a variety of assistance scenarios that range, e.g., from giving reminders for medications to monitoring of Activity of Daily Living, from giving advices to promote an healthy lifestyle to psychological monitoring. Among possible users, frail older adults deserve a special focus as they present a rich variability in terms of both alternative possible assistive scenarios (e.g., hospital or domestic environments) and caring needs that could change over time according to their health conditions. In this perspective, robot behaviors should be customized according to properly designed *user models*. One of the long-term research goals for SAR is the realization of robots capable of, on the one hand, *personalizing* assistance according to different health-related conditions/states of users and, on the other, *adapting* behaviors according to heterogeneous contexts as well as changing/evolving needs of users. This work proposes a solution based on a user model grounded on the international classification of functioning, disability and health (ICF) and a novel control architecture inspired by the dual-process theory. The proposed approach is general and can be deployed in many different scenarios. In this paper, we focus on a social robot in charge of the synthesis of personalized training sessions for the cognitive stimulation of older adults, customizing the adaptive verbal behavior according to the characteristics of the users and to their dynamic reactions when interacting. Evaluations with a restricted number of users show good usability of the system, a general positive attitude of users and the ability of the system to capture users personality so as to adapt the content accordingly during the verbal interaction.

## Introduction

Socially Assistive Robotics (SAR) aims at designing robots capable of ensuring social interaction to human users in a variety of assistance scenarios that range, e.g., from giving reminders for medications to monitoring of Activity of Daily Living, from giving advice to promote an healthy lifestyle to psychological monitoring (Feil-Seifer et al. 2005; Tapus et al. 2007), as well as allowing a significant reduction in caregivers burden (Shukla et al. 2017). Among other services, the possibility to exploit SAR as supportive technology for delivering cognitive training represents an interesting opportunity, since cognitive disability is one of major aging’s challenges (Yuan et al. 2021). Hurtado et al. describe a platform able to deliver cognitive training services (Hurtado et al. 2021). More in detail, the games, designed for cognitive stimulation based on the items of the Mini Mental State Examination (MMSE), are exploited in a bi-modal way of interacting (speech and a touch screen interface). In Shukla et al. (2017), a robot was developed to provide training by covering all the main cognitive functions, like temporal orientation, attention, gnosis and perception, memory, executive functions, calculus and language. In this work, the robot was able to deliver the training both complementing the therapists or even conducting the sessions autonomously. Besides being able to act autonomously, SAR might represent a valuable support to the therapists for personalizing assistance to different users’ needs (Sorrentino et al. 2022; Di Napoli et al. 2022). There is evidence that cognitive games delivered on a robot may be a valuable addition to existing cognitive stimulation activities and that the robot can be considered easy to use and useful in improving cognitive functioning (Gasteiger et al. 2021; Hurtado et al. 2021). Nevertheless, a crucial aspect during the interaction between the robot and the user is the ability to establish a continuous, personalised, credible and engaging relationship (Yuan et al. 2021).

With this in mind, the authors are pursuing a line of work in SAR where the key goal is to realize a general *cognitive control approach* capable of supporting continuous assistive behaviors, personalized and adapted to the different and evolving needs of patients (Umbrico et al. 2020, 2021; De Benedictis et al. 2020).^1^ The need of implementing forms of “intelligent behaviors” in social robots requires to investigate a research direction that leads to the integration of Robotics and Artificial Intelligence (AI) (Lemaignan et al. 2017; Cortellessa et al. 2021; Ingrand and Ghallab 2017). This integration is especially crucial to support *personalized* and *adaptive* social and assistive interactions with humans. It is indeed necessary to *customize* general interaction capabilities of robotic platforms to the specific features of the interaction scenario (e.g., hospitals, private houses for SAR), preferences and health-related needs of users (Moro et al. 2018; Rossi et al. 2017). In this regard, an “expressive” and well-structured *user model* is fundamental to realize effective human–robot interactions. On the one hand, it allows robots to *personalize* their general interaction/assistive capabilities (i.e., *behaviors*) to the specific needs and features of users. On the other hand, it allows robots to *adapt* behavior execution over time according to the changing or evolving states of users (e.g., worsening of impairments, changing health-related needs or changing interaction preferences etc.).

This work specifically focuses on the *user model* defined to represent both health-related features and needs of persons as well as their interaction characteristics and preferences (e.g., users general attitudes and preferences). The *user model* is based on the *International Classification of Functioning and Disabilities* (ICF) proposed by the World Health Organization (WHO).^2^ The model includes variables that characterize *stable* or *slow-changing* aspects of a user (e.g., functioning of short-term memory or sustaining attention) as well as *dynamic* and *fast-changing* aspects of a user (e.g., the current mood of a patient) that may change even during interactions and thus requiring a higher degree of adaptation. Slow-changing variables of the model in particular allow a robot to *personalize* assistive behaviors by identifying the sub-set of robotic services (e.g., cognitive stimulation, physical stimulation, therapy reminders, health parameter monitoring etc.) that best fit the specific impairments of a user (i.e., health-related needs). These variables allow a robot to reason about “what” services a particular user actually needs. Fast-changing variables of the model instead allow a robot to *adapt* the execution of assistive behaviors to the actual (contextual) state of a user (e.g., user mood). These variables allow a robot to reason about “how” identified services should be executed in order to be effective.

The developed model is used within a novel cognitive architecture inspired by the *dual-process theory* (Kahneman 2003) which entails two reasoning layers working (continuously and simultaneously) at different abstraction levels and making contextualized and integrated decisions over different temporal horizons (e.g., hours/days for the *slow reasoning layer* and seconds/minutes for the *fast reasoning layer*). The motivations behind this work is the long-term objective of realizing an advanced cognitive control architecture capable of endowing SAR systems with the “general” reasoning and interacting capabilities necessary to support various scenarios (e.g., domestic daily assistance, in-hospital rehabilitation support), heterogeneous needs (e.g., therapy reminders, cognitive stimulation, monitoring of health parameters) and different preferences and features of patients (e.g., different modalities of interactions, daily schedules). Although this work specifically focuses on the administration of personalized cognitive stimulation programs, the scope of the methodology and proposed technological approach is broader and concerns the capability of *tailoring* general SAR services to heterogeneous assistive scenarios taking into account different user needs and clinical objectives (Umbrico et al. 2021, 2020).

The two-layers are realized integrating reasoning modules based on heterogeneous AI technologies to support the cognitive capabilities needed at the different levels of abstraction. A *slow-reasoning layer* uses a pipeline of *Knowledge Representation & Reasoning* (KRR) and *Automated Planning* (AP) modules to represent high-level human knowledge and generate *stimulation strategies* encapsulating a general vision about the specific needs of the assisted person. A *fast-reasoning layer* uses a policy-based approach to execute step-by-step an interaction strategy combining speech acts tailored to the assisted human. It is worth observing that taken alone, each of these technologies (and layers) would not completely support the desired requirements. As an example, AP (Rajan and Saffiotti 2017; Ingrand and Ghallab 2017) is well suited to synthesize a complex set of actions supporting the desired assistive objectives but, it lacks of the flexibility and adaptability needed to naturally interact with humans. Similarly, KRR (Tenorth and Beetz 2017; Jansen and Schulz 2011; Guarino 1998) is well suited to represent the domain features of assistive scenarios and support contextualized reasoning but, it lacks of a “runtime perspective” and it is not suited to directly deal with the unpredictable behavior of the interactions with a human. On a different perspective, policies generated by *Reinforcement Learning* (RL) approaches (Sutton and Barto 2018) can have the reactivity levels and the adaptation capabilities desired to efficiently deal with human behavior dynamics but, they lack of a general “long-term goal-oriented perspective.” Furthermore, RL lacks of the “semantics” and the “explicit structures” needed to *explain* behaviors of robots to humans (Došilovic et al. 2018). The achievement of a such a policy through the learning process, however, inherently depends on the size of the state space which, to manage high-level and/or temporal aspects, grows significantly.

After some related works (Sect. 2), the paper presents the two layered architecture (Sect. 3), describes the user model (Sect. 4), the reasoning layer (Sect. 5) and the interactive layer (Sect. 6), respectively. Section 7 presents our current architecture at work and discusses how certain behavior has been obtained. Section 8 describes a preliminary evaluation with users while Sect. 9 ends the paper envisaging limitations and future work.

## User models for adaptive human–robot interaction

When interacting with the external world, human beings rely on mental models that are assumed to be internal symbols or representations of external reality, hypothesized to play a major role in cognition, reasoning and decision-making. This concept, firstly introduced by the psychologist Kenneth Craik Kenneth (1943), is widely used in the human–computer interaction field through the efforts directed to the user modeling research that describes the process of building up and modifying a conceptual “understanding” of the user. The main goal of *user modeling* is indeed the customization and adaptation of the systems’ behavior to the user’s specific needs and preferences. For this purpose, a social robot needs an internal representation of the user. User modeling has a long lasting tradition in research and this brief section does not pretend to present an exhaustive presentation of related work. Rather it gives a short overview of the works that are closer to our idea of user modeling for SAR.

In Fong et al. (2003), the authors highlight how social robots should be able to perceive and understand the richness and complexity of natural human social behavior in order to interact with people in a socially acceptable way. Detecting and recognizing human action and communication is an excellent basis for adapting robot behavior appropriately. A key mechanism for enabling this ability is in fact reasoning over a user model. In this sense, user models can be used for a variety of purposes. First, they can help the robot to represent and understand the human behavior and adapt the interaction accordingly. Dialogues, for example, can be adapted and contextualized to a specific situation thanks to a user model. Secondly, based on a user model, robot’s movements (gesture, body position, gaze direction, etc.) and the pace of interaction (e.g. knowing when to insert pauses) can be appropriately guided by users’ profiles. Finally, user models are useful for adapting the robot behavior to suit users with different skills, experience and knowledge.

A recent survey (Rossi et al. 2017) highlights how the development of robotic systems capable of modeling and correctly recognizing human behavior and adapting their functioning with respect to the user is a very critical task. This is especially true in the domain of assistive robotics where robots interact with a vulnerable user population. The review proposes a classification of works related to user modeling and adaptation of robot behaviors taking into account three viewpoints: (i) *Physical*, considering all the aspects related to the human body; (ii) *Cognitive*, related to the capability of inferring and recognizing, the intentions, belief, internal states, personality and emotions; (iii) *Social*, related to the social signals displayed by the users.

There are several factors that can be considered for user modeling and that, consequently, can influence the generation of possible different behaviors. For example, Tapus et al. (2008) describes a socially assisted robot therapist designed to monitor, assist, encourage and socially interact with post-stroke users engaged in rehabilitation exercises. The work investigates the role of the robot’s personality in the therapy process, focusing on the relationship between extroversion-introversion level of the robot and the personality traits of a user. The reported results demonstrate how the adaptation of the autonomous behavior of the social assistive robot to the user’s personality can lead to an improvement in the performance of human tasks. Another example of the usefulness of user models can be found in Yixing Gao et al. (2015). In this work, the authors propose a method based on real-time upper-body pose and user models to plan robot motions in a scenario of personalised assistance through a dressing application for users who have upper-body movement limitations. In this case, the user model is based on four pre-defined sets of goal positions which are sent successively to the robot to execute, where exact values of these goal positions are determined dynamically according to real-time human upper-body pose and user models.

In general, the creation of suitable user models for fostering human–robot interaction serves the ultimate goal of reaching an interaction characterized by: fluent behaviors, adaptability, trust building, effective communication and explainability. In Tabrez et al. (2020), the authors categorize the methods for mental modeling in human–robot interaction contexts by organizing them into three categories: (i) first-order models where robots model the behavior of human collaborators to infer their beliefs, intentions, and goals for purpose of predicting their actions; (ii) second-order models, related to a recursive type of reasoning one step deeper in behavior modeling, by enabling robots to possess more predictable and explicable behavior, as the effects of their actions on the other agent’s perception of them are included in the model; (iii) shared models aimed at establishing shared understanding and common knowledge as a basis for selecting actions that are consistent and coordinated with those of the counterpart.

Differently, in Hiatt et al. (2017), the authors discuss and compare the many techniques available for modeling human cognition and behavior and provide a classification categorizing these techniques according to the Marr’s levels (Marr 1982) of analysis, namely the computational level, the algorithmic level and the implementation one. Following these three levels, the categorization aims at describing the many techniques used for achieving robust human–robot collaboration where robots should understand what humans do in general (computational level), how they do it (algorithmic level) and how cognition is physically realized (implementation level).

The mentioned works represent some examples of the literature that has focused on one or more aspects of human–robot interaction and have proposed the use of AI technologies to realize dynamic and adaptive behaviors. It is worth observing that a “standard user model” for SAR is missing. This is in part due to the high variability of application scenarios and in part to the large number of “user-related variables” that may affect assistance and implemented interactions. As a result, researchers have typically defined their “own” user model focusing on the specific aspects that were relevant in the considered contexts. One of the objectives of our work is to investigate the definition of a sufficiently general model capable of supporting the representation of a wide variety of information that directly or indirectly may affect robotic assistance. To this aim, it was natural for us to build our model on the International Classification of Functioning, Disabilities and Health. This classification represents a *standard framework* to characterize the level of functioning of a person from different perspectives. It is therefore specifically suited to characterize *health-related needs* of a user determining *what* kind of assistance is needed (e.g., cognitive and physical impairments) but also *personal* and *environmental features* determining *how* assistance should be carried out to be effective (e.g., gender, age, cultural aspects and other factors).

## Layered approach to user modeling and adaptive behavior

The user model is the shared component of the designed control approach. It encapsulates the *knowledge* an assistive robot needs to autonomously recognize the health-related needs of a user, the assistive objectives that should be pursued and how such objectives should be achieved in order for the assistance to be effective. Our long-term research objective is to realize an AI-based control approach capable of endowing robots with the *cognitive capabilities* necessary to support continuous, personalized and adaptive assistance (Umbrico et al. 2021). Following our research experience (Cortellessa et al. 2021), the approach and the underlying user model should be sufficiently general to support different scenarios ranging from general daily-living assistance to specialized in-hospital assistance. The particular *shape* of supported assistance clearly depends on the specific needs of the involved users and the assistive context. The model should therefore characterize all the features necessary to synthesize services that are relevant/useful to a user as well as identify the conditions under which such services should be carried out in order to be effective.

In this sense, the ICF classification represents a wide and quite complete theoretical framework. It is well suited to characterize users from different but synergistic perspectives. In fact, ICF considers both functioning and disability as complex interactions between the individual’s health conditions and environmental and personal factors. The classification considers them as dynamic aspects in interaction with each other and modifiable over time. Based on this classification, we propose a user model focused on four main dimensions: *Physical*, which is related to body functions and structures; *Psychological*, that is linked to mental functions both cognitive and emotional; *Social*, that is related to relationship and attitudes toward others and; *Environmental*, that is related to the physical context and means of the interaction.

It is also important to point out that the components of the user model (and the underlying variables) are not static but may change over time. These variables capture heterogeneous aspects of a user that can change with different “time scales.” This means that some parts of the user profile may change slowly while others may change more rapidly.

Aspects like those concerning physical or cognitive impairments for example would change in the “long-term” and can thus be considered static during the execution of one or more assistive tasks (e.g., a daily assistive plan). Aspects like those concerning emotions or user mood, instead, can change quickly. These parts of the user profile can be considered *dynamic*, requiring a fast adaptation of the robot’s behaviors (even within the execution of a particular assistive task). Such changes specifically have effects on the way a robot interacts with a user and thus on the way assistance is carried out in the “short-term.” Similarly, the model determines different ways a robot acquires information about users and thus the way variables composing user profiles are set and updated. Variables concerning slow-changing aspects of a user, e.g., physical or cognitive impairments are set by taking into account input from clinicians. In particular, we consider the contribution of standard screening procedures in order to directly integrate knowledge from clinicians. As shown in Sorrentino et al. (2022) for example the outcome of the MMSE can be map to ICF variables and used to *initialize* a user profile with validated data about his/her cognitive state. Variables concerning fast-changing aspects of a user e.g., user mood or emotions are instead dynamically set by the robot while interacting with a user. A robot would, for example, start interacting with a user knowing nothing about his/her mood. As the execution of the assistive tasks goes on, then the robot refines the user profile by inferring his/her current mood, emotions and other aspects. According to this information, the robot would, for example, adapt the *interaction style* in order to realize a more effective and engaging assistance.

**Fig. 1:**
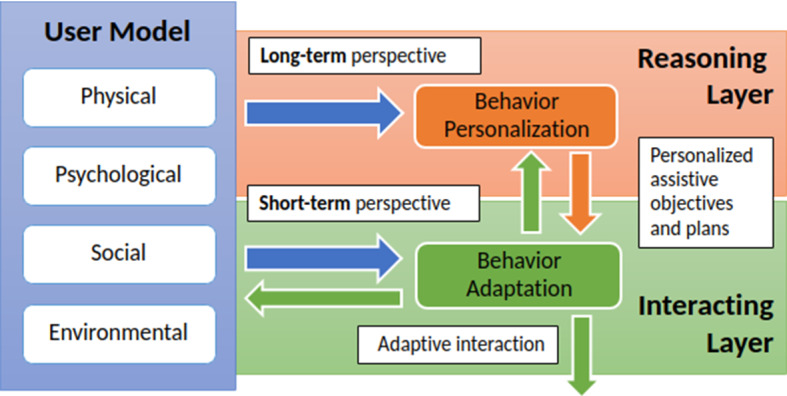
A comprehensive view of the proposed approach. The User Model influences both the reasoning and the interacting layer (blue arrows). The reasoning layer sends high-level commands to the interacting layer (orange arrow) which provides feedback, in terms of successes and failures, to the reasoning layer (green upward arrow). Finally, the interacting layer produces actions toward the environment (green downward arrow) and, along interacting with the user, update the model (green arrow pointing to the left)

To effectively deal with these dynamics, we pursue the integration of different reasoning processes that support online dynamic and natural interactions while maintaining an updated long-term perspective of the assistive objectives to be pursued. Figure 1 shows the proposed two-layered system integrating these short- and long-term perspectives. The long-term perspective mainly focuses on *personalization* and realizes the reasoning processes that are necessary to identify health-needs of a user, assistive objectives and accordingly contextualize the needed assistive services. The short-term perspective instead mainly focuses on *adaptation* and realizes the reactive processes that allow the robot to dynamically adapt the way assistance is executed taking into account the evolving state of a user and feedback.

The *reasoning layer* focuses on the *long-term perspective* and thus mainly relies on the variables of the user model that “change slowly” (e.g., changes in the cognitive and physical functioning). This layer in particular relies on symbolic AI techniques to endow a robot with the cognitive capabilities necessary to *recognize* health conditions of users, *decide* coherent assistive objectives as well as autonomously *decide* contextualized assistive actions that would achieve such objectives. The *interacting layer* instead focuses on the *short-term perspective* and thus mainly focuses on the variables of the user model that “change rapidly” (e.g., emotion, environmental conditions). This layer is specifically designed to support a natural interaction with the user (e.g., through state-of-the-art *Natural Language Processing* techniques) and integrates *policy-based* AI techniques to decide how to concretely interact with a user and promptly react to unexpected human behaviors and environmental change. This layer specifically supports natural conversations by generating adaptive *dialogue-based interactions* between the assistive robot and the user.

A critical aspect about SAR consists in the ability to comprehend and generate human-like interactive behaviors. Ideally, such robots should cooperate with and be taught by non-humans, so that they can be applied in a wide range of contexts with ease. An initial list of desiderata, which although not exhaustive might serve as a good starting point for discussing the state of the art, is presented in Mavridis (2015). The combination of the two layers, in particular, allows to move toward a “purposeful speech and planning.” While classical approaches to automated planning tends to be very effective in achieving goal-oriented behaviors, they result are poorly suited for an effective human–robot interaction, as a consequence of the high uncertainty due to factors ranging from the imperfections of current speech processing systems to the unpredictability of behavior of the user. Approaches based on Partially Observable Markov Decision Processes (POMDP) are able to manage this uncertainty, but lose the ability to do goal-oriented reasoning.

By combining slow and fast reasoning layers, the proposed approach mitigates the above limitations by delegating uncertainty management to the interacting layer and goal-oriented behavior to the reasoning one. Once learned, offline, how to classify the user’s intents, and define the corresponding actions in the reactive layer, the system becomes able to manage multiple speech acts. Moreover, thanks to the introduction of the planner, it is possible to plan interventions by the system, allowing a mixed initiative interaction. The combination of these two capabilities allows to overcome the “simple commands only” barrier. Furthermore, the interaction with the user is personalized based on an estimate of the user’s profile and his current mood, allowing the adoption of an affective interaction. Finally, although not extensively covered in this document, our approach considers forms of non-verbal communication (see, for example, Fig. 6, for an intuition of how the interactive layer’s actions have effects on the facial expressions of the robot).

## An ICF-based framework for user modeling

The design of a complete and sufficiently general *user model* is crucial to support *user awareness* and allow SAR systems to *tailor* general assistive services to the specific needs and features of target users (LeRouge et al. 2013). Other works in the literature have investigated the design of effective user models pursuing user-centered design (Gena and Weibelzahl 2007). However, a widely recognized model capable of capturing health-related needs of users for SAR applications is still missing. For example, the work (Heckmann et al. 2005) introduces the ontology GUMO designed to support uniform representation of users among different user-adaptive systems. The model proposes a taxonomical description of a number of features, e.g., *user personality*, *emotional state* or *mental state* that would be useful to characterize users in SAR applications. Although general, GUMO does not allow to “quantify” the level of functioning of physical and cognitive features of a users and thus would not support fine-grained reasoning about the *level of impairment* of users. Furthermore, GUMO only focuses on users and thus would not support representation of robot assistive capabilities as well as reasoning about suitable assistive actions. A user model for SAR applications in our opinion should take into account a “clinical perspective” and capture user-related knowledge in a formalism that is easily understandable by clinicians and possibly compliant with existing clinical standards.

Our investigation led to the identification of ICF as the theoretical framework to ground a user model for SAR. Broadly speaking, the ICF classification characterizes the ability of a person of using her/his physical/cognitive skills (i.e., *level of functioning*) taking into account both “internal” physical factors and “external” environmental factors. It allows, for example, to interpret *mobility* of a person according to both the intrinsic ability of coordinating his/her muscles and using motor skills (levels of physical impairment) and the features of the surrounding environment (e.g., presence of physical barriers and obstacles). This framework therefore supports a quite flexible representation and interpretation of health-related knowledge of users. Furthermore, it is a “standard” widely known by practitioners and would facilitate *knowledge sharing* and *communication* between clinicians and SAR systems.

Other works have used ICF to characterize cognitive and physical conditions of users. For example, the work (Kostavelis et al. 2019) introduces a novel robot-based assessment methodology of users’ skills to characterize the needed level of daily assistance. The work (Filippeschi et al. 2018) uses ICF to characterize cognitive and physical skills of users and accordingly represent the outcomes of the implemented robot-based assessment procedures. Similarly, the work (García-Betances et al. 2016) uses ICF to represent needs and requirements of different types of user and support a user-centered design of ICT technologies. In particular, this work integrates an ontological model of ICF into the cognitive architecture ACT-R (Anderson et al. 1997) to simulate the behaviors of different types of user. Nevertheless, these works present a “rigid” and static representation as they usually do not rely on a well-structured ontological formalism to dynamically contextualize *knowledge* about users (i.e., *user profiles*) in different situations. Such works usually do not integrate online reasoning mechanisms to allow assistive robots to *autonomously reason* about the specific needs of a user and autonomously (or partially autonomously) *decide* the kind of intervention that best fit such needs. Conversely, our approach pursues a highly flexible solution implementing the cognitive capabilities needed to *understand* health conditions of users and s(autonomously) personalize assistance accordingly, under the supervision of a human expert.

The defined *user model* and the developed representation and reasoning capabilities rely on a *domain ontology* (Guarino 1998). The use of ontologies is typical to enhance cognitive capabilities of robots and allow artificial agents to, for example, autonomously evaluate *opportunity* of interactions with the environment (Beßler et al. 2018; Tenorth and Beetz 2017), support *behavioral qualities* like, e.g., self and context *awareness* (Awaad et al. 2015; Bruno et al. 2019; Borgo et al. 2019), or *behavior flexibility* and *proactivity* (Lemaignan et al. 2017; Umbrico et al. 2021). Ontological models and knowledge-based reasoning capabilities therefore well support personalization and adaptation of robot behaviors. In Umbrico et al. (2020), we developed a first ICF-based ontological model to support adaptive daily-living assistance. The model was deployed into a cognitive architecture called KOaLa (*Knowledge-based cOntinuous Loop*) and was effective in tailoring daily-living monitoring services of SAR to the specific needs, preferences and known routines of patients. KOaLa constitutes the reasoning layer of the approach depicted in Fig. 1 and here is further extended in order to enhance reasoning and personalization capabilities. More specifically, we extend the ontological framework with concepts and properties that allow a SAR system to *know* (and reason about) the *effects* that (some) assistive actions like, e.g., the administration of a cognitive exercise, may have on the health state of a person.

This work extends KOaLa by introducing the capability of reasoning about such effects and thus reason about possible *actions* that can be performed to (theoretically) *alter* the health state of a target users. We here specifically focus on cognitive stimulation to show how the added semantics features enhance the flexibility of robot behaviors in terms of *what* kind of services a user needs and *how* such services should be carried out to be effective. The domain ontology (TBox), the knowledge base (ABox) and related reasoning capabilities have been developed using standard semantic technologies (OWL Antoniou and van Harmelen (2004), Apache Jena^3^) and off-the-shelf tools (Protégé^4^). Table 1 describes the “macro-dimensions” of the ICF-based user model and their correlations with *personalization* and *adaptation* capabilities of a SAR system.

**Table 1:** Dimensions of the user model

User model	Personalization	Adaptation
*Physical features*		
Hearing functions and structures Visual functions and structures Speech functions and structures	To determine the general interaction modalities used by a robot to communicate with a user during the assistive task. For example, a robot could prefer using voice-based over text-based interactions in case of users with impaired visual functions	To determine the way a robot physically interact with a user and the environment and, the way it reacts to user feedback. In case of users with impaired hearing functions, for example, a robot could decide to move closer to her when reproducing some audio message
*Psychological Features*		
Personality Emotions Attention Memory Psyco-motor functions	To determine the type of assistance a user needs and the desirable objectives to pursue in the long-term. For example, users with memory and/or attention impairments would need a constant stimulation	To determine the “communication strategy” of a robot. Depending on the specific personality traits of a user, for example, a robot can change the way it reacts to positive and negative feedback (e.g., correct or wrong answers to cognitive exercises)
*Social features*		
Communication capabilities Interpersonal interaction and relation capabilities Social participation	To enrich the assistance services with information related to the social context of the user (e.g., a user could be part of a specific association and the system could enrich the plan with meaningful information related to this activity)	To determine the communication style of the robot in accordance with the ability of the person to understand verbal and/or non-verbal signals
*Environmental features*		
Presence of Assistive Devices Cultural context Characteristics of the physical environment	To enrich the assistance services, for example, by taking into account the cultural context of the user (religion, Country, traditions, etc.) that could influence his/her attitude toward others	To adapt the robot according to dynamic changes in the environment (e.g., automatic change of screen brightness in case of decreased luminosity of the room)

Four main dimensions are considered. The *Physical dimension* concerns the physiological and structural functioning of human body. Some examples can be related to the sensory functions such as hearing and seeing taking into account both physical and physiological impairments. The *Psychological dimension* concerns all those characteristics that can be associated to the mental functioning from both a cognitive (e.g., attention and memory) and emotional (e.g., mood) point of view. The *Social dimension* concerns the capabilities of a person of producing and receiving messages in both verbal and non-verbal modalities, as well as their relationships and participation in social contexts. The last *Environmental dimension* then concerns the contextual aspects, such as culture, physical environment and the presence of assistive devices (e.g., glasses). Sections 5 and 6 further explain which variables of the user model (and how) are used, respectively, by the reasoning and interacting layers of Fig. 1.

### Representing health needs of users

The semantic model of a user should formally characterize all the information a robot needs to *represent* and *reason* about the health state of an assisted person. Following Table 1, the ontology relies on the “reification” of some ICF dimensions to form a well structured and scientific basis for the description of health and context-related states. The ontological model relies on the foundational ontology DOLCE (Gangemi et al. 2002) as theoretical background for the integration of the ICF dimensions.

In this regard, the concept DOLCE:Quality is used to interpret ICF concepts as *functioning qualities* characterizing cognitive and/or physical aspects of a person. First we have defined the concept FunctioningQuality as a specialization of DOLCE:Quality with the aim of characterizing general functioning aspects of a DOLCE:Person. We have integrated the OWL ICF taxonomy officially distributed by WHO^5^ as specialization of FunctioningQuality to completely support the ICF framework. The concept FunctioningQuality represents the *root element* of the defined taxonomy of *functioning qualities* of a DOLCE:Person. This root element is defined as *equivalent* to the concept WHO:ICFCategory of the ICF taxonomy and it is then further specialized into a number of ICF concepts like, e.g., Attention, Memory or Calculation that describe different aspects of the “functioning” of a person, at different levels of abstraction (e.g., the concept Memory can be further specialized into Short-term Memory, Long-term Memory and so on).

To measure functioning qualities, we extend the concept DOLCE:Region. According to DOLCE, this concept models any dimensional space which can be used as a value for a measured quality of an entity of the domain. We have thus defined the concept FunctioningRegion as a specialization of DOLCE:Region in order to characterize a dimensional space to FunctioningQuality of a DOLCE:Person. We have defined the concept FunctioningRegion as *equivalent* to the concept WHO:Performance that us a sub-class of ICF Qualifier of the integrated ICF taxonomy. The resulting dimensional space thus consists of 6 concepts (subclasses of FunctioningRegion/WHO:Performance) that comply with the measuring interval of the ICF scale: (i) the value 0 denotes NoImpairment (no difficulty, 0–4%); (ii) the value 1 denotes SoftImpairment (mild difficulty, 5–24%); (iii) the value 2 denotes MediumImpairment (moderate difficulty, 25–49%); (iv) the value 3 denotes SeriousImpairment (severe difficulty, 50–95%); (v) the value 4 denotes a FullImpairment (complete difficulty, 96–100%) and; (vi) the values 5 and 6 represent the impossibility of measuring a quality (not specified and not applicable, respectively).

### Reasoning on impairments and interaction modalities

Given the functioning qualities and the associated dimensional space, it is necessary to define concepts that describe the specific health state of a user at a particular point in time. To this aim, we have defined the concept Profile as sub-concept of DOLCE:Description. It represents a “descriptive context” of the functioning qualities of a DOLCE:Person. A *profile* is composed by a number of Measurement (specialization of DOLCE:Diagnosis). Each individual of Measure associates an individual of FunctioningQuality to an individual of FunctioningRegion, expressing the outcome of the measurement within the ICF scale. A user profile instance can thus be seen as a Knowledge Graph (KG) (Ehrlinger and Wöß 2016) associating an instance of DOLCE:Person to a set of *values* each of which measures a specific functioning quality of a user.

1 $$\begin{aligned} \begin{array}{r l} \texttt {Profile} \sqsubseteq &{} \texttt {Description} \sqcap \\ &{} \exists \texttt {describes}.\texttt {Person} \sqcap \\ &{} \exists \texttt {hasPart}.\texttt {Measure} \end{array} \end{aligned}$$

2 $$\begin{aligned} \begin{array}{r l} \texttt {Measure} \sqsubseteq &{} \texttt {Diagnosis} \sqcap \\ &{} \exists \texttt {hasConstituent}.\texttt {Person} \sqcap \\ &{} \exists \texttt {isRelatedTo}.\texttt {Profile} \sqcap \\ &{} \exists \texttt {measures}.\texttt {FunctioningQuality} \sqcap \\ &{} \exists \texttt {outcome}.\texttt {FunctioningRegion} \end{array} \end{aligned}$$

Each score of a profile denotes the *level of impairment* of a specific functioning quality of an assisted person. A profile can be processed in order to automatically *infer* impairments and characterize the cognitive state of a person. Given the measured functioning qualities of a user an assistive robot is thus autonomously capable of identifying aspects that need assistance and recognize *situations of impairment*. We have defined the concept Impairment as specialization of DOLCE:Situation. According to the semantics of DOLCE:Situation, an Impairment represents a *view* on the Profile of an DOLCE:Person *satisfying* a number of conditions on FunctioningQuality. Impairment situations are thus *inferred* through the following rule:

3 $$\begin{aligned} \begin{array}{r l} \forall x,y,w,z. \exists i. (\texttt {Measurement(x)} \wedge &{} \\ \texttt {measures(x,y)} \wedge &{}\\ \texttt {hasConstituent(x,w)} \wedge &{}\\ \texttt {FunctioningQuality(y)} \wedge &{}\\ \texttt {Person(w)} \wedge &{}\\ \texttt {hasOutcome(x,z)} \wedge &{}\\ \texttt {hasICFScore(z)} > 0 \wedge &{}\\ \texttt {hasICFScore(z)} < 4 \rightarrow &{} \texttt {Impairment(i)} \wedge \\ &{} \texttt {concerns(i,w)} \wedge \\ &{} \texttt {concerns(i,y)} \wedge \\ &{} \texttt {satisfies(i,x)}) \end{array} \end{aligned}$$

Rule 3 defines as Impairment any *situation* where the “measured outcome” of a FunctioningQuality (i.e., hasICFScore(z)) is characterized as a *soft* (i.e., score 1), *medium* (i.e., score 2) or *serious* impairment (i.e., score 3). The rule excludes from impairments those situations where measured qualities are too seriously compromised (i.e., score 5) since they require dedicated treatments that are supported by SAR systems usually. Considering for example the case of *cognitive stimulation* and assuming a therapist provides as input the ICF scores characterizing the (assessed) health state of a patient (i.e., her/his profile), the defined concepts and rules allow an assistive robot to (autonomously) analyze the profile of a DOLCE:Person and *infer* the impairments that should be addressed to properly stimulate her/his cognitive state.

It is worth noticing that a Profile and inferred Impairment encapsulate *knowledge * useful to characterize the *interaction abilities* of a DOLCE:Person also. For example, if the analysis of a profile infers a*medium impairment* of the quality Hearing then, the interactions between the user and the robot should rely mainly on visual and textual messages rather than voice and audio. In case that audio interactions cannot be avoided (e.g., recorded audio instructions and recommendations or videoconferences) it would be possible to properly set the sound level of the robot in order to help the assisted person as much as possible. In addition to *impairments*, *interaction parameters* can be (autonomously) inferred by a robot in order to determine *how* assistance should be carried out. Specifically, we have considered four interaction parameters: (i) *sound level*; (ii) *subtitles*; (iii) *font size*; (iv) *explanation*.

The parameter *sound level* has values {*none*, *regular*, *high*} and specifies the volume of audio communications from the robot to the user. Users with soft or medium impairment of Hearing would need a high sound level. Similarly audio would be completely excluded for persons with serious impairments in order to use different interaction modalities. The parameter *subtitles* has values {*none*, *yes*, *no*} and specifies the need of supporting audio messages through text. Users with soft or no impairment of Seeing and medium or serious impairment of Hearing would need subtitles to better understand instructions and messages from the robot. The parameter *font size* has values {*regular*, *large*} and specifies the size of the font of text messages and subtitles, if used. Users with medium impairment of Seeing would need *large* fonts in text messages in order to better read their content. Finally, the parameter *explanation* has values *yes* or *no* and specifies the need of explaining an exercise to a user before its execution. Such instructions would be particularly needed for users with impaired Memory or Orientation. Clearly, the way such explanations are carried out complies with the interaction parameters described above.

### Contextualizing assistive capabilities

In order to properly reason about possible actions composing assistive behaviors, the ontological model should characterize the *assistive capabilities* of a robot (and a SAR system in general). A formal representation of the *effects* that assistive actions may have on the health state of patients (i.e., on the set of FunctioningQuality of a DOLCE:Person) is necessary. On the one hand, it is necessary to characterize general *assistive actions* in terms of *stimuli* pursuing a *functional representation* (i.e., describing assistive actions in terms of their effects on health states). On the other hand, it is necessary to correlate *stimuli* to *user profiles* in order to reason about the *relevance* of different stimuli with respect to the specific *impairments* of a person. Such semantics is crucial to dynamically contextualize *known* robot capabilities with respect to the specific health needs of an assisted person.

To this aim, we follow a semantics similar to the *Taxonomy of Functions* introduced for manufacturing domains (Borgo et al. 2014, 2009). We specifically pursue the conceptual view of *functions* as “qualitative descriptions of actions” characterizing *effects* they have on the *qualities* of domain entities. A key aspect of this *interpretation* is the separation between the physical features and operations that “implement” some conceptual actions and the “external features” of these actions characterizing their relationships with the entities of a domain.

4 $$\begin{aligned} \begin{array}{r l} \texttt {StimulationFunction} \sqsubseteq &{} \texttt {Method} \sqcap \\ &{} \exists \texttt {isPartOf}.\texttt {Stimulus} \sqcap \\ &{} \exists \texttt {hasEffectOn}.\texttt {FuncQuality} \end{array} \end{aligned}$$

**Fig. 2:**
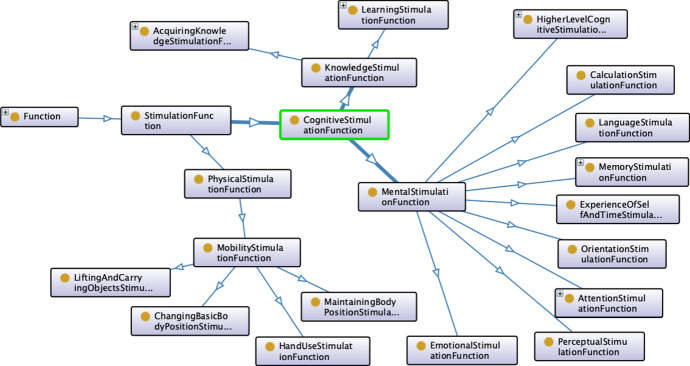
Excerpt of the taxonomy of stimulation functions

This separation supports *modularity* of an ontological model and *flexibility* of reasoning mechanisms. It allows to deal with any concrete type of action a robot can perform by “simply” reasoning on its *effects*. Following this interpretation, we define the concept of StimulationFunction to characterize the *effects* of known stimuli on the FunctioningQuality of a DOLCE:Person. More specifically, we define a StimulationFunction as a particular type of DOLCE:Method describing procedures that have some *effects* on FunctioningQuality of a DOLCE:Person. Concerning the cognitive stimulation scenario, each *administration action* of a cognitive exercise (i.e., individuals of Stimulus) is described by the associated set of StimulationFunction which in turn characterizes the effects on the FunctioningQuality of a DOLCE:Person. Figure 2 shows an excerpt of the defined taxonomy of StimulationFunction.

A key aspect to point out is the 1:1 mapping between the taxonomy of StimulationFunction and the taxonomy of FunctioningQuality. This design choice supports all possible granularity levels for representing and reasoning about the effects of functions. According to the specified association is thus possible either to reason at the lowest level of detail considering the elements of StimulationFunctionthat are associated to the leaves of the taxonomy of FunctioningQuality or to reason at a higher level of detail considering functions that are associated to intermediate elements of the taxonomy and thus aggregating multiple FunctioningQuality. These two taxonomies share the same theoretical background based on the ICF classification which is crucial to support *contextual reasoning* and link stimulation actions to user profiles.

More in detail, *contextual reasoning* leverages the concept of *affordances* that has been defined by Gibson as “opportunities for actions” (Gibson 1977). Although Gibson’s definition concerns mainly opportunities of actions “enabled” by objects, we here “extend” this concept in order to characterize *opportunities of stimulation* enabled by the capabilities of an assistive robot (Umbrico et al. 2020). In Robotics, the concept of affordances has been used and refined by many researchers with the aim of improving the *flexibility* of robot behaviors (Beßler et al. 2020; Bozcuoğlu et al. 2019; Awaad et al. 2015). An affordance characterizes a relational concept contextualizing properties of objects with skills and capabilities of robots to dynamically infer actions (opportunities) that can be performed in a particular scenario.

This flexible interpretation well generalizes to opportunities of stimulation. We thus define the concept of Affordances as a particular type of DOLCE:Role in order to emphasize the pursued relational semantics. We define the concept StimulationOpportunity as a specific type of Affordances correlating exactly one impairment situation and exactly one stimulation function of an assistive robot supported by some stimulus.

5 $$\begin{aligned} \begin{array}{r l} \texttt {StimOpportunity} \sqsubseteq &{} \texttt {Affordances} \sqcap \\ &{} \exists ! \texttt {classifies}.\texttt {Impairment} \sqcap \\ &{} \exists ! \texttt {classifies}.\texttt {StimFunction} \end{array} \end{aligned}$$

Stimulation opportunities are dynamically interpreted according to the inferred impairments of a user and to the actual (stimulation) capabilities of a robot. An assistive robot can thus dynamically infer the set of stimuli (and related stimulation actions) that enable *stimulation opportunities* and “can afford” the impairments of a patient. This general schema is formally described by the logic rule below inferring the set of StimulationOpportunity.

6 $$\begin{aligned} \begin{array}{r l} \forall x,y,w,z. \exists o. (\texttt {Impairment(x)} \wedge &{} \\ \texttt {FuncQuality(y)} \wedge &{}\\ \texttt {concerns(x,y)} \wedge &{}\\ \texttt {StimFunction(w)} \wedge &{}\\ \texttt {hasEffectOn(w,y)} \wedge &{}\\ \texttt {isPartOf(w,z)} \wedge &{}\\ \texttt {Stimulation(z)} \rightarrow &{} \texttt {StimOpportunity(o)} \wedge \\ &{} \texttt {classifies(o,x)} \wedge \\ &{} \texttt {isRelatedTo(o,y)} \wedge \\ &{} \texttt {isRelatedTo(o,w)} \wedge \\ &{} \texttt {canAfford(z,x)}) \end{array} \end{aligned}$$

## Personalization through planning

Given a user profile and a set of inferred situation opportunities, a *personalized assistive plan* should be defined by taking into account the set of stimuli that “best” address the impairments of a person. This section describes the knowledge reasoning mechanisms developed to *rank* infer stimuli and extract *recommendations* then formalized as a planning problem to synthesize suitable assistive behaviors.

### From user profiles to stimulation recommendations

Given a user profile there can be several stimulation opportunities that would be inferred and potentially a significant number of stimuli and stimulation actions that would address the impairments of a person. It is therefore necessary to realize *ranking mechanisms* that identify the *most relevant stimuli* and use this knowledge to personalize assistance. A “semantic-based” recommendation process enriches the reasoning layer of Fig. 1 bridging *user knowledge* with *planning knowledge* through the extraction of contextualized stimuli. The relationships between the inferred stimuli and the ICF-based functioning qualities of the taxonomy are encoded by an *incidence matrix* $A_{m,n}$ where: (i) columns matrix are associated to the *n* elements of the functioning qualities of the taxonomy; (ii) rows are associated to the set of *m* stimuli extracted from the inferred stimulation opportunities. A value of the matrix $A(i,j) = 1$ denotes that the *i*-th stimulus *can afford* the functioning quality represented by the j-th element of the taxonomy. A value of the matrix $A(i,j) = 0$ instead denotes that the *i*-th stimulus *cannot afford* the functioning quality represented by the j-th element the taxonomy.

Let us consider *k* distinct user profiles stored into the knowledge base of an assistive robot. Knowledge about these profiles can be represented as a *profile matrix*. Each element of the matrix $V(i,j) \in \ [0, 4]$ characterizes the functioning level of the *i*-th quality of the taxonomy with respect to the j-th profile of the knowledge base. Since both matrices rely on the ICF-based taxonomy of functioning qualities, it can be observed that the number of columns of the matrix $A_{m,n}$ is equal to the number of rows of the profile matrix $V_{n,k}$. We can thus combine the incidence matrix $A_{m,n}$ with the profile matrix $V_{n,k}$ in order to obtain a *ranking matrix* $R_{m,k}$ expressing a number of recommendations. A value $R(i,j) \in \ {\mathbb {R}}_{0}^{+}$ of the ranking matrix specifies a *rank* denoting the “relevance” of the *i*-th known stimulus to the j-th stored profile. The higher the rank the more the stimulus is relevant for a particular profile.

Without loss of generality, we can consider the particular case where only one profile is stored into the knowledge base of an assistive robot. In this case, the equation below computes a *ranking vector* $R_m$ (i.e., a ranking matrix $R_{m,k}$ where $k = 1$) representing the “relevance” of known stimuli. The higher the value $r_i$, the higher the *relevance* of the *i*-th stimulus with respect to the *impairments* of a person.

7 $$\begin{aligned} \begin{aligned} R_{m,1} = A_{m,n} \times V_{n,1} = \begin{bmatrix} a_{1,1} &{}... &{} a_{1,n}\\ a_{2,1} &{}... &{} a_{2,n}\\ ... &{}... &{}...\\ a_{m,1} &{}... &{} a_{m,n} \end{bmatrix} \begin{bmatrix} v_1\\ v_2\\ ...\\ v_n \end{bmatrix} \\ = \begin{bmatrix} a_{1,1}v_1 +... + a_{1,n}v_n\\ a_{2,1}v_1 +... + a_{2,n}v_n\\ ...\\ a_{m,1}v_1 +... + a_{m,n}v_n \end{bmatrix} = \begin{bmatrix} r_1\\ r_2\\ ...\\ r_m \end{bmatrix} \end{aligned} \end{aligned}$$

### Personalized stimulation as a planning problem

Assistive plans are generated by taking into account all health-related needs of a patient (e.g., monitoring of physiological parameters, reminders about medical appointments and dietary restrictions etc.), technical requirements (e.g., battery constraints of the robotic platform) and preferences (Umbrico et al. 2020). Focusing on the *cognitive stimulation problem*, generated recommendations are given to a planner as input in order to synthesize a *personalized stimulation plan*. We specifically consider plans that daily stimulate the cognitive state of a person. Plans are therefore synthesized daily, scheduling the administration of (recommended) exercises during specified *interaction windows* (i.e., *user preferences*). The planning model ${\mathcal {S}}_M$ of a *cognitive stimulation problem* is characterized by a number of parameters that define *known* interaction preferences of a patient, measured functioning qualities (i.e.. her/his *profile*) and a number of stimulation capabilities of a robot. Such *planning knowledge* can be formalized as follows:

8 $$\begin{aligned} {\mathcal {S}}_M = (W_u, F_u, m_f, Ex, afford_{ex}, effect_{ex}) \end{aligned}$$

where $W_u$ is the set of *interaction windows* characterizing the *interaction preferences* of a user. Each window $w_i \in W_u$ is defined as the following tuple:

9 $$\begin{aligned} w_i = (s_i, d_i, x_i, v^{l}_i, v^{s}_i, t_i) \end{aligned}$$

where: (i) $s_i \in {\mathbb {T}}$ is the start-time of the window; (ii) $d_i \in {\mathbb {Z}}$ is the duration of the interaction window; (iii) $x_i \in \{true, false\}$ is a boolean variable denoting the need of *explaining* an exercise; (iv) $v^{l}_i \in \{none, low, medium, high\}$ denotes the preferred sound level of audio messages; (v) $v^{s}_i \in \{true, false\}$ is a boolean variable denoting the use of subtitles and; (vi) $t_i \in \{none, normal, large\}$ denotes the preferred font size of text messages/instructions.

$F_u$ is the set of *functions* describing the “cognitive functioning” of the assisted person. Each symbol $f_i \in F_u$ denotes a specific cognitive capability of the ICF-based profile of the user. The function $m_f$ denotes the *measurements* of these functions $f_i$ acquired during the *profiling phase* of the user.

10 $$\begin{aligned} m_f: F_u \rightarrow \ {\mathbb {R}}^{+}_{0} \end{aligned}$$

For each (cognitive) function $f_i \in F_u$ of the ICF-based profile of a user, the function $m_f$ returns a positive value $m_f(f_i) = v \in {\mathbb {R}}_{0}^{+}$ representing the *outcome* of the profiling procedure. The outcomes of the evaluation and therefore the values of the functions are $m_f(f_i) \in \ \{0, 1, 2, 3, 4\}$.

*Ex* is a set of symbols denoting exercises the robot should consider for the synthesis of stimulation actions. The *capabilities* of these exercises are modeled by means of the functions $afford_{ex}$ and $effect_{ex}$.

11 $$\begin{aligned} afford_{ex}: Ex \rightarrow \ F \end{aligned}$$

The function $afford_{ex}$ associates each exercise $e \in Ex$ with subset $F_{e} \subseteq \ F$ of (cognitive) functions the exercise *e* *can afford*.

12 $$\begin{aligned} effect_{ex}: Ex \times F \rightarrow [0,1) \end{aligned}$$

The function $effect_{ex}$ denotes the *effects* that the administration of an exercise $e_i \in Ex$ have on a function $f_i \in F$ of a user. We assume that the administration of an exercise to a user has some *positive* effect on his/her cognitive state with respect to the functioning qualities the exercise can actually afford, i.e., $f_i \in afford_{ex}\left( e_i\right)$. The function $effect_{ex}$ can thus be further defined as

13 $$\begin{aligned} effect_{ex}(e_i, f_i) = \left\{ \begin{array}{@{}ll@{}} 0, &{} \text {if} f_i \not \in afford_{ex}\left( e_i\right) \\ (0, 1), &{} \text {otherwise}\\ \end{array}\right. \end{aligned}$$

A problem instance ${\mathcal {S}}_P$ defines the *initial situation* characterizing the starting cognitive state of the user, the *goal situation* characterizing the desired cognitive state of the user and a *deadline* characterizing the duration of the plan. The cognitive state of a user is defined as the set $I_u$ of functions $f_i \in F_u$ whose measurement denotes some level of impairment.

14 $$\begin{aligned} I_u = \{f_i \in F_u: m_f\left( f_i\right) > 0\} \end{aligned}$$

An *initial situation* of a planning problem ${\mathcal {S}}_P$ is therefore defined as the set of impairments at the beginning of a plan (i.e., $I^{t}_{u}$ where time $t = 0$). A *goal situation* similarly is defined as the set of *desired* impairments at the end of the execution of a plan (i.e., $I^{t}_{u}$ where time $t = H$),

15 $$\begin{aligned} {\mathcal {S}}_P = (I^{0}_{u}, I^{H}_{u} = \emptyset , H) \end{aligned}$$

where $H \in {\mathbb {T}}$ represents the deadline of the current stimulation cycle and therefore it constrains the duration of stimulation plans (i.e., the *plan horizon*).

Given a planning model ${\mathcal {S}}_M$ and a planning problem ${\mathcal {S}}_P$, a planning process can synthesize *stimulation plans* $\Pi$. A plan $\Pi = \left( {\mathcal {A}}, {\mathcal {S}}\right)$ is defined by a set of stimulation actions ${\mathcal {A}}$ and a *scheduling function* ${\mathcal {S}}: {\mathcal {A}} \rightarrow \ W_u$, constraining actions $a_i \in {\mathcal {A}}$ to occur *during* one of the available interaction windows $w_i \in W_u$. Each action $a_i \in {\mathcal {A}}$ of the plan represents a request dispatched to the reactive layer of Fig. 1 for the actual administration of a certain exercise $e_i \in Ex$, following the specified interaction preferences. The interaction parameters of an action $a_i \in {\mathcal {A}}$ should be *merged* with the interaction parameters of the associated interaction window $w_i$.

16 $$\begin{aligned} a_i = (e_i, x_i, v^{l}_{i}, v^{s}_{i}, t_i) \end{aligned}$$

The integrated AP capabilities rely on a timeline-based framework called PLATINUm (Umbrico et al. 2017). A timeline-based model is composed of a set of *state variables* describing possible temporal behaviors of domain features. Each state variable consists of a number of *values* representing states or actions the related feature may assume or perform over time. Each *value* is associated with a *flexible duration* and a *controllability tag* which specifies whether the value is controllable or not. A *state transition function* specifies valid sequences of values of a state variable. Additional constraints among different state variables can be specified by means of *synchronization rules*. Following a modeling approach early proposed in Cesta et al. (2014), two main state variables have been defined for the considered planning problem. One state variable models the stimulation capabilities of an assistive robot (Eq. 16). The other state variable models the interaction windows and preferences of the user (Eq. 9). Examples and features of stimulation plans are given in Sect. 7 to show the whole personalization process “in action”.

## Adaptation through policy-based interactions

In this section, we describe the second layer our architecture can count on. The need for such a module is triggered by the requirement for a continuous and reactive interaction that is very relevant in a non-deterministic environment. The presence of a real user in the environment, and its naturally unpredictable behavior, requires continuous and rapid adaptations of the generated plans. Such adaptations, in particular, is hard to support by the Reasoning Layer (Fig. 1) that, for its nature, adopts more farsighted yet slower forms of reasoning. As a consequence, those aspects related to the direct interaction with the users are delegated to the Interacting Layer (same Figure) which, adopting a control-based approach, is able to respond to unpredictable user behavior.

**Fig. 3:**
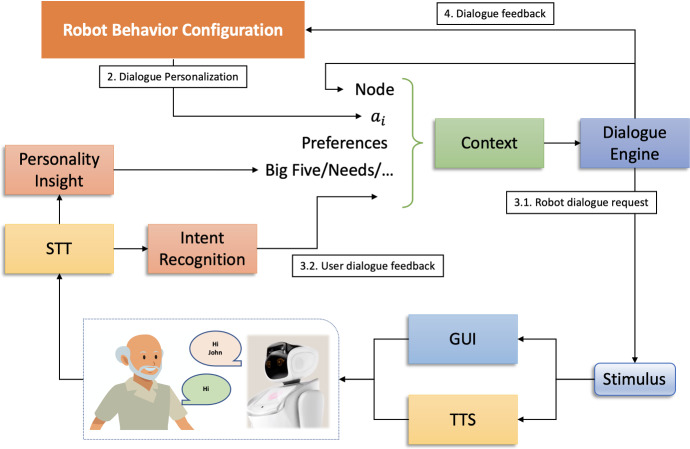
Internal components of the reactive reasoner

Unlike the reasoning layer described in the previous section, which by carrying out higher level forms of reasoning synthesizes a sequence of high-level actions to be executed over time, the role of the interacting layer, sketched in Fig. 3, consists in selecting actions based on a *context* that dynamically evolves over time. In other words, the objective of the reactive module consists in adopting a *policy* $\pi \left( ctx \right) = a$ which, given a context *ctx*, returns an action *a* to be executed. The execution of such actions, in particular, physically translates, in our case, in the pronunciation of personalized sentences in natural language toward a user. Since, however, these actions internally constitute transitions in a *state-transition system* (Dean et al. 1991), we propose a new form of parametric actions, inspired by those used in classical planning (Ghallab et al. 2004), which allow to represent the state-transition system in an implicit and more compact way. Before describing these actions, however, it is advisable to define the states of such a state-transition system which, by analogy with natural language generation systems, we will call *context* (Montenegro et al. 2019; Herbert and Kang 2018).

From a technical perspective, we refer by *context* to a set of variables, both symbolic and numeric, which characterize the current state of the system. These variables, in particular, numerically characterize some of the dimensions described in Table 1 (e.g., physical features, psychological features like mood and personality, etc.), enriching them with further components used internally by the interacting layer in order to make the interaction with the user more natural. More specifically, context variables are used to keep track of all the information that, more or less dynamically, change over time, including all those factors which are relevant for the discrimination of the actions taken by the system such as, for example, those related to the user’s personality and current mood as well as those elements related to the interaction like, for example, information extracted from the user’s speech analysis and the robot’s facial expressions (refer to Table 2 for a more detailed description of some of the used context variables which are more relevant for the interacting layer). By dynamically updating the values of the context variables, the system, through the adopted policy, will adaptively select actions with the aim of personalizing the interaction and obtaining dialogues and, more in general, behaviors, that are as fluid and engaging as possible.

**Table 2:** The context variables used by the reactive reasoning module with their initial value and a brief description

Name	Init value	Description
Intent	None	Used for representing the user’s intents. Values are set with the results of the NLU module
n	None	A numeric variable used for recognizing numbers in the users’ utterances. Values are set with the results of the NLU module
Sentiment	0	A numeric variable ranging from − 1 to 1 representing the sentiment, from negative to positive, of the last utterance from the user. Values are set with the results of the NLU module
Extraversion	None	A numeric variable ranging from 0 to 1 representing the extraversion of a user. Values are set with the results of the personality insight module
Node	None	Used for representing the current node of the state-transition system of the exercise. Values are set by executing actions
Num_errors	0	Used for representing the current performance of the user in terms of the number of errors made. Values are set by executing actions
Sound_level	Regular	Used for adapting the audio volume for persons with hearing impairments. Values are set by the reasoning layer through high-level actions
Subtitles	None	Used for specifying the need of supporting audio messages with text. Values are set by the reasoning layer through high-level actions
Font_size	Regular	Used for specifying the size of the font of text messages and subtitles. Values are set by the reasoning layer through high-level actions
Exercise	None	Used for describing the current rehabilitation exercise. Values are set by the reasoning layer through high-level actions

The updating of the context variables, in particular, might take place as a consequence of different events such as: (a) the transmission and interpretation of high-level commands coming from the execution of the customized plans produced by the reasoning layer; (b) as a consequence of the effects of the actions performed by the dialogue engine (more details about this case later); and (c) as a consequence of the changes that the environment adopts regardless of the behavior of the robot. More specifically, the latter case constitutes the main motivation for lightening the weight of the reasoning layer by introducing the interacting layer. Context variables, in particular, are also modified as a consequence of the unpredictable interactions that the user has with the system.

### Grasping the intentions of the users

Since the reactive module interfaces with state-of-the-art Natural Language Understanding (NLU) modules,^6^ it is appropriate to introduce some basic terminology and to summarize a few concepts to better understand the proposed architecture. A first distinction in NLU, indeed, is the one between the *utterance*, that is anything the user says, like, for example, “How did I fare with memory games yesterday?”, and the user’s *intent*, that is a characterization of the intention the user was aiming at in issuing the utterance. Each intent, specifically, is identified by a name which can be used for reasoning upon it. As an example, since the intention of a user saying to the robot something like “How did I fare with memory games yesterday?” is to request for some information (namely, the performances of “yesterday” in “memory games”), the name associated to the intent can be, for instance, #askPerformance. Additionally, intents can be further characterized by *entities* (also called, in some cases, *slots*), representing possible modifiers for the intent, aiming at enriching the agent’s understanding capabilities. Entities, in particular, can be fields, data, or text describing just about anything (e.g., a time, a place, a person, a number, etc.). Likewise the intents, entities are identified by a name which characterizes its typology and can be used for reasoning upon them. As an example, the words “yesterday” and “memory” might be associated to two entities, whose names might be respectively @date and @game, which enrich the #askPerformance intent by specifying that the user is seeking for the performance of a specific game typology performed at a specific time.

By providing a set of utterance examples, each characterized by an intent and, possibly, a set of entities, an expert user can train an NLU system to recognize the users’ intents and, possibly, the presence of entities, from a users’ utterance which is not necessarily contained within the training set. Suppose, for example, that the user says something like “How was my performance yesterday at the memory games?”, the NLU module matches the utterance with the best available intent and entities returning, at the same time, a *confidence* value associated with the classification (e.g., confidence 0.9 for a #askPerformance intent along with a value “03/03/2021” for the @date entity and a value “memory” for the @game entity, assuming that the utterance is pronounced on 04/03/2021). This process, referred to as *intent recognition* or *intent classification*, aims at making generic sentences, pronounced by users, understandable and manageable by an autonomous system.

Specifically, by exploiting state-of-the-art modules, the interacting layer analyzes data coming from the sensors (in particular, the signal from a microphone) and, through a speech-to-text module^7^ translates the user’s vocal utterance into text. Subsequently, through the NLU module, the interacting layer analyzes the recognized text for extracting high-level information (specifically, intent, entities and the confidence value) and assigns the resulting values to the corresponding context variables.

### A compact description of the dialogue engine’s policy

Depending on all the values of the context variables, whenever any of them is updated, the interacting layer invokes the policy for the selection of the next *action* to execute, producing a contextualized response for the user and possibly making further changes to some of the current values of the context variables. Actions, in particular, are selected based on how they are defined. More specifically, actions are characterized by three elements: (a) a logical combination (i.e., conjunctions and/or disjunctions) of *conditions* on the context variables for establishing the executability of the action; (b) a set of natural language sentences representing the system’s *responses* provided to the users whenever the action is executed (if the set contains more than one sentence, one is chosen randomly); and (c) a set of *effects* on the context variables, representing the updates to apply on both symbolic and numeric context variables whenever the action is executed.

Each action whose conditions are verified in the current context is said to be *executable*. Whenever asked to the system, as a consequence of the interactions from a user or for the starting of a high-level action from the reasoning layer, all executable actions are executed in the order they are defined by the expert user. The presence of such actions, indeed, is intended both to establish which responses to provide to the users and to make further transitions in the context space by means of the actions’ effects updating, for example, some context variables about the current discussion topic. Similarly to classical planning, actions are compactly described by means of first-order *operators* which, all together, constitute a compact representation of the interaction policy.

Finally, some of the operators can be linked to more complex behaviors in case the actions subsumed by them require, for example, the access to a database or the invocation of a REST service. As an example, the aim of a tell_performance(?date, ?game) operator might be to respond to the user whenever the robot is questioned for some information about the performance for a specific game played in a specific date. Additionally, this operator is characterized by a ?date parameter, indicating the date related to the requested information, and a ?game parameter indicating the game typology of the requested information. Furthermore, the operator is characterized by the intent == #askPerformance condition, which triggers the execution of the action in case the #askPerformance value is assigned to the intent parameter. Finally, the operator has, as responses, the sentences “In date ?*date* you made $?num\_errors$ errors in ?*game* games.” and “You made $?num\_errors$ errors in ?*game* games.”.

Suppose, as an example, that the user asks to the robot “How did I fare with memory games yesterday?”. The utterance is translated into text from the speech-to-text module and the NLU system recognizes the #askPerformance intent along with the “03/03/2021” value for the @date entity and a “memory” value for the @game entity. After assigning the #askPerformance value to the intent parameter, the “03/03/2021” value for the date parameter and the “memory” value for the game parameter, the interacting layer seeks for all the executable operators. When it comes to the tell_performance(?date, ?game) operator, since its execution condition is satisfied, the system recognizes the operator as executable, randomly selects one of the associated responses and replaces all the occurrences of the operator parameters with the corresponding values taken from the context variables, thus composing a contextualized response for the user. However, in the specific case of the tell_performance(?date, ?game) operator, requiring an access to a database to generate a correct answer, the performance for the “memory” game in date “03/03/2021” is searched in the database among the stored past played games and the query’s result, suppose is “4”, is assigned to the num_errors context parameter, just before performing the parameter substitution. The final result, therefore, is either the answer “In date 03/03/2021 you made 4 errors in memory games,” or the answer “You made 4 errors in memory games.”

Note that the value of context variables remains persistent until it is replaced by a new one. In case of the ?date parameter, for example, the “03/03/2021” value can be exploited in case of a second question from the user, asking about another game typology with a sentence like “What about the attention games?”, without specifying any date and assuming, like in a natural talk, that the user is actually referring to the attention games played on “03/03/2021.”

In conclusion, by ignoring in their conditions the values of all the irrelevant context variables to generate a specific response, the organization of the actions into operators allows to significantly compact the description of the state transition function. Thanks to the proposed approach we are able to answer questions such as the one on the memory game’s performance introduced above, made *during* the execution of a cognitive exercise, without losing the focus on the exercise, as long as certain limits, that depend on the unpredictable behavior of the user (and, clearly, on the defined operators), are respected. Finally, although the definition of the operators is, at the moment, an operation that is performed manually by an expert user at system programming stage, it is worth noticing that its introduction allows to easily correct, in a debugging stage, any problems in the interaction with the users.

### Personality insight and sentiment analysis

An interesting aspect of the interactive module that is worth highlighting is its ability to customize its behavior according to the user’s personality. The idea is to give the reactive module a personality similar to that of the user with whom it interacts. The similarity-attraction principle (Folkes 1982), indeed, assumes that individuals are more attracted to others who manifest the same characteristics. Indeed, it has been suggested that interpersonal similarity and attraction are multidimensional constructs in which people are attracted to people similar to themselves in demographics, physical appearance, attitudes, interpersonal style, social and cultural background, personality, preferred interests and activities, communication and social skills (Lydon et al. 1988).

By having available all the past utterances collected from the interactions with the user, in fact, we can exploit them to feed a state-of-the-art module of personality insight^8^ which, based on the psychology of language in combination with data analytics algorithms, is able to infer personality characteristics such as the Big Five personality traits (i.e., openness to experience, conscientiousness, extraversion, agreeableness and neuroticism) starting from a text provided by the user. The text from which personality is recognized, specifically, is, in our case, given by the collected past utterances. Taking inspiration from Tapus and Mataric (2008), the extroversion-introversion trait is taken into account for modulating the robot’s behavior which will act by using more reassuring and nurturing words in case of introverted persons, and with more challenging words in case of more extroverted persons. More in detail, whenever a user says something, the utterance is appended to the old ones, forming the text for the personality recognition module. The recognized extraversion value updates the “extraversion” context variable, whose value influences the executable actions actions.

The last aspect worth being considered concerns the customization of the system behavior based on the current mood of the user. Once again we rely on state-of-the-art tools^9^ for analyzing the sentiment of the user’s last utterance so that we can estimate his/her mood and customize the behavior of the system accordingly. Also in this case the recognized sentiment updates the “sentiment” context variable, whose value influences the executable actions actions by means of the defined executability conditions.

## The case study of cognitive stimulation

This section describes a runtime validation of the proposed approach aiming at showing the implemented reasoning processes. Section 7.1 shows contextual reasoning and personalization by taking into account a number of realistic user profiles. A first part shows how the system is capable of dynamically identifying different needs and evaluating the relevance of a set of cognitive exercises, according to the user’s profile. It then shows an example of personalized stimulation plan dynamically synthesized according to the inferred knowledge about users’ needs stimulation recommendations. Section 7.2 shows how a personalized stimulation plan is executed by actually administrating cognitive exercises to a user. Specifically, we show how the dialogue-based interaction module adapts the interaction to user feedback and dynamically extracted/inferred contextual (dialogue) knowledge.

### Contextual reasoning and personalization

To support runtime validation, we have defined three real user profiles (*U1*, *U2* and *U3*) whose ICF scores are shown in Table 3. The considered profiles resulted from a clinical assessment made by a clinician during the cognitive evaluation of some patients. Consequently, in this evaluation, we focus on a sub-set of ICF mental functions accordingly to the deficits obtained from the assessment. As can be seen from Table 3, we consider *attention function*, *memory function*, *perceptual function* and *higher-level cognitive function*.

**Table 3:** User profiles for runtime assessment

ICF variables		U1	U2	U3
b140 Attention functions	b1400 Sustaining attention	1	3	2
	b1401 Shifting attention	0	2	3
	b1440 Short-term memory	1	3	1
b144 Memory functions	b1441 Long-term memory	2	3	3
	b1442 Retrieval and processing	0	2	3
	b1443 Working memory	1	1	3
b156 Perceptual functions	b1560 Auditory perception	0	3	1
	b1561 Visual perception	1	1	3
b164 Higher-level cognition functions	b1640 Abstraction	2	3	1
	b1641 Organization and planning	1	2	3
	b1643 Cognitive flexibility	0	2	1

In addition, we define *domain knowledge* about *cognitive exercises* that can be actually considered in cognitive stimulation scenarios. Based on the literature and with the support of experts in the field of cognitive rehabilitation, these exercises were chosen, since they can be administered through vocal channel, while maintaining the capability to work on different cognitive functions. Table 4 shows the list of these exercises and the functioning qualities they support. For example, exercise *B* (*recall words*) can be used to stimulate *Short-term Memory* (b1440) and *Retrieval and Processing* (b1442). Similarly, exercise *F* (*word classification*) can be used to stimulate *Sustaining Attention* (b1400), *Retrieval and Processing* (b1442) and *Working Memory* (b1443).

**Table 4:** Stimulation capabilities of considered cognitive exercises

Exercise	Short description	Capabilities
A	Recall a short story	b1441 - Long-term memory b1442 - Retrieval and processing
B	Recall words	b1440 - Short-term memory b1442 - Retrieval and processing
C	Recall list of things	b1440 - Short-term memory b1443 - Working memory
D	Find the word	b1440 - Short-term memory b1442 - Retrieval and processing
E	Read a story	b1440 - Short-term memory b1442 - Retrieval and processing
F	Word classification	b1440 - Short-term memory b1442 - Retrieval and processing b1443 - Working memory
G	Idiom	b1440 - Short-term memory b1640 - Abstraction
H	Planning	b1641 - Organization and planning
I	Split task	b1401 - Shifting attention b1643 - Cognitive flexibility

**Fig. 4:**
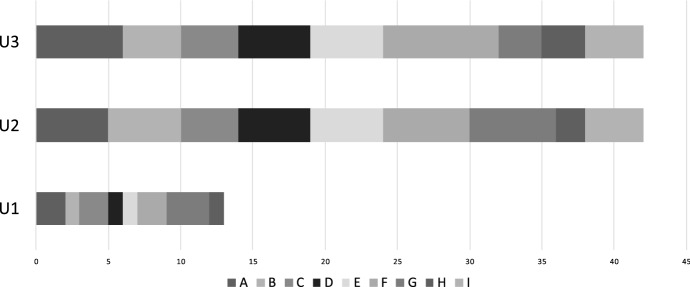
Overall impairment status of users and inferred relevance of exercises

Figure 4 shows the outcome of knowledge reasoning and the *inferred rankings* of modeled exercises with respect to the profiles of *U1*, *U2* and *U3*. As can be seen, the relevance of the exercises and related stimulation actions changes significantly according to the different needs of a user. User *U3* for example has several medium and serious impairments of *Memory Function* and *Attention Function* (b1400, b1441, b1442 and b1443). Knowledge reasoning infers therefore that exercise *F* is strongly relevant to *U3*. Actually *F* is the most relevant exercise for *U3* since it addresses many of the impaired qualities of the user. Conversely, exercise *G* for example addresses *Short-term Memory* and *Abstraction* that are two of the most impaired qualities of user *U2*. This exercise is therefore inferred as particularly significant for *U2*.

This knowledge is then used to formalize a planning problem and synthesize personalized stimulation plans that are executed through dialogue functionalities. For example, a timeline-based stimulation plan for *U3* will consider exercises *A*, *D*, *E* and *F* while a timeline-based stimulation plan for *U2* will consider exercises *B*, *D*, *E*, *F* and *G*. Let us consider with more detail a personalized plan for *U3* with three *interaction windows* described below, according to Eq. 9.

17 $$\begin{aligned} \begin{aligned} w_1 = (5, 5, \text {no}, \text {high}, \text {yes}, \text {large})\\ w_2 = (25, 15, \text {no}, \text {high}, \text {yes}, \text {large})\\ w_3 = (45, 35, \text {yes}, \text {high}, \text {yes}, \text {large}) \end{aligned} \end{aligned}$$

Each interaction window defines a possible schedule of stimulation actions and characterizes interaction preferences. The window $w_2$ for example starts at $s_1= 25$ time units from the origin of the plan and has a duration of $d_1=15$ minutes. Interaction parameters specify that *U3* does not require explanations (the first parameter $x_2 = no$) while requires a high sound level of the robot (the second parameter $v_2^l = \text {high}$), the use of subtitles (the third parameter $v_2^s = \text {yes}$) and a large font size (the fourth parameter $t_2 = \text {large}$).

Planned stimulation actions should fulfill both time and interaction requirements. The code below shows three stimulation actions that would compose the timeline of the robot as defined by Eq. 16:

18 $$\begin{aligned} \begin{aligned} a_1 = (D, \text {no}, \text {high}, \text {yes}, \text {large})\\ a_2 = (F, \text {no}, \text {high}, \text {yes}, \text {large})\\ a_3 = (E, \text {yes}, \text {high}, \text {yes}, \text {large}) \end{aligned} \end{aligned}$$

with schedules ${\mathcal {S}}(a_1) = w_2$, ${\mathcal {S}}(a_2) = w_2$ and ${\mathcal {S}}(a_3) = w_3$.

### Adaptive administration of cognitive exercises

Suppose now that the dialogue-based assistance module receives the command to start the action $a_1$. This event corresponds to the starting of the administration of the cognitive exercise *D* (refer to Table 4) to a user who has no cognitive impairment, who, because of age, does not hear very well. For this reason, the stimuli must be delivered at a high volume with a regular speed and, since she/he also does not see very well, the font on the robot screen must have a large size. The cognitive exercise, the volume, the speech speed and the font size, therefore, enrich the context of the interacting layer along with the information about the preferences and the personality of the user. Based on these parameters, the module, through the policy, will select the most suitable concrete action to present to the user.

**Fig. 5:**
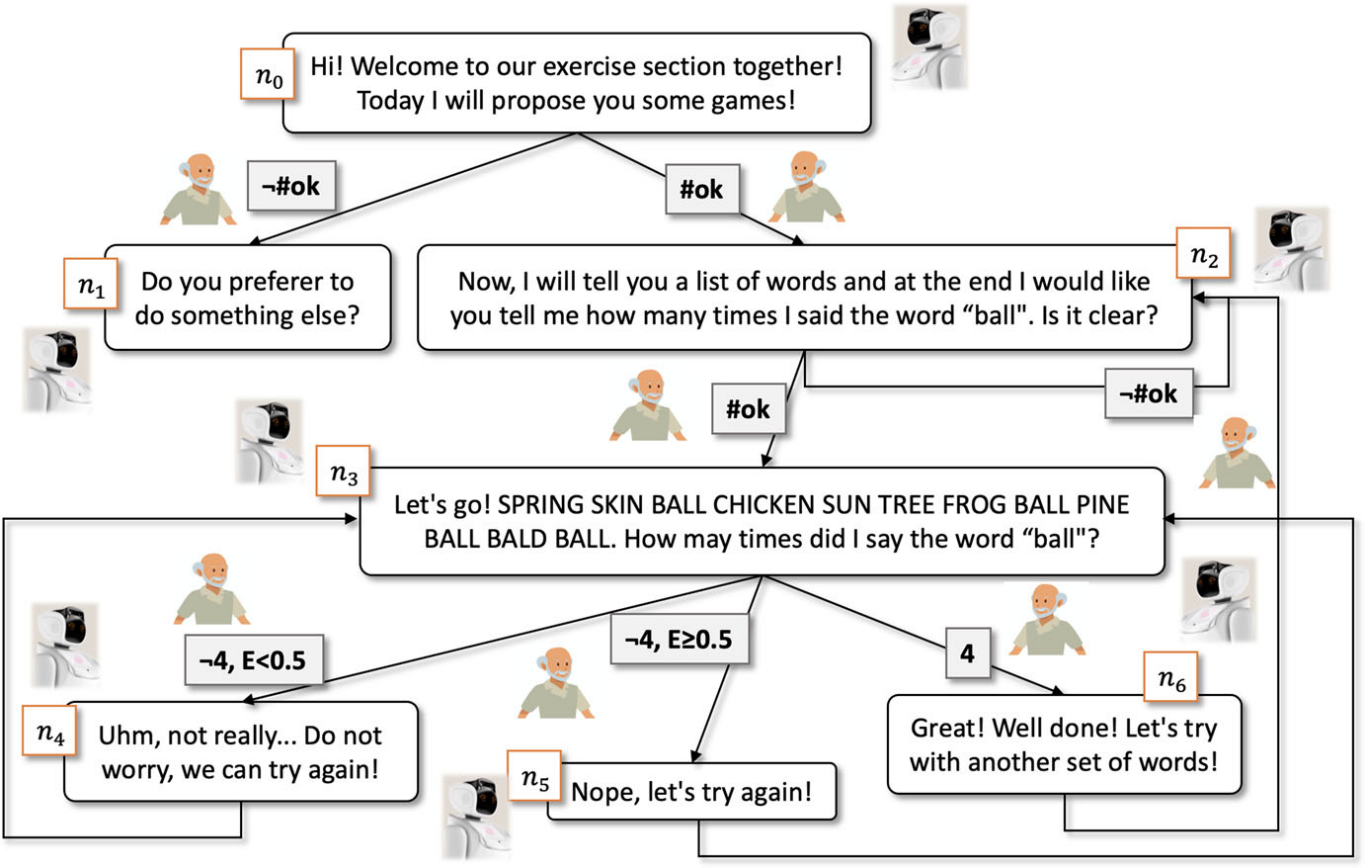
Simplified dialogue scheme for exercise *D* “Find the word”

In particular, the cognitive exercise *D*, called “Find the word,” consists in sending a series of words while asking to pay attention to one specific word. At the end of the list, the robot asks to the user how many times the target word has been said. The robot starts a conversation as described in Fig. 5 by introducing the exercise section. The user answers, for example, with a sentence like “OK, let’s start!” which is recognized by the intent recognition module as an $\#ok$ intent. At this point, the interacting layer invokes the policy for the selection of the actions to be executed, providing a description of the exercise to the user, through the responses of the selected operators and, through their effects, assigning the value $n_2$ to the *node* context variable. It is worth noting that the reasoning layer could not predict the user’s response and, therefore, could not include a consequent response in the initial plan. In the absence of the interacting layer, in particular, the reaction to the user’s response would have required a potentially expensive adaptation of the plan.

At this point, the user answers affirmatively and the exercise continues with the robot listing a set of words to the user and asking him how many occurrences there are of a specific word (in the example, ball”). The interesting aspect is that the responses that the interacting layer provides to the user might depend on various factors that characterize the context. In particular, in the event of an incorrect answer, the system can respond more reassuringly, in case of an introverted person (i.e., the value of the Extroversion context variable assumes a value which is less than 0.5), or in a more challenging way, in case of a more extroverted person (i.e., the Extroversion context variable value is greater than 0.5). Along with personalizing the responses, in case of incorrect answers the interacting layer increases the value of the num_errors context variable, so as to allow a second operator to detect a number of errors above a certain threshold and communicate to the reasoning layer the failure of the cognitive exercise and the need to generate a new plan which is more suited to the person’s abilities. Finally, whether with a failure or with a success, at the end of the exercise the performance is saved on a local database both for allowing the user to ask for information on the past performance, as in the example of Sect. 6.2, and for generating statistics which can be consulted by a healthcare professional.

Through this example, it has been demonstrated the capability of the developed system to administer tailored training programs which dynamically adapts to the occurring situations. Basically, starting from a tailored configuration of the training plans, through the verbal interaction with the user, the robot has been able to change the plans execution in order to adapt to the user’s contingent needs with the ultimate goal of completing the exercise by maintaining the user engaged.

In the following section, a preliminary implementation of the proposed solution has been tested by involving real users in laboratory setting.

## User involvement for solution validation

The example illustrated in Sect.  7.2 was used to carry out an experimental session involving representative users. This validation represents a preliminary step before involving frail users. In fact, in this period of pandemic crisis it was particularly difficult to think of an assessment involving older adults since face-to-face sessions could be risky. For this reason, we have opted for a preliminary assessment involving a small number of healthy adults, always in compliance with anti-COVID19 regulations, confident of being able to subsequently organize a more complete assessment in safe conditions with frail elderly people. For this reason, the following validation focuses more on the assessment of technical and performance on metrics rather than its efficacy as training tool. For this assessment, the above-mentioned exercise “Find the word” was chosen as a task in an experimental session in order to evaluate both usability of the solution and its capability to personalize its interaction during the exercise unfolding.

### Participants

Thirteen participants were involved in the evaluation session. More in detail, 8 males and 5 females with an average age of 48.46 (SD= 14.3) participated. As general information, they were asked to express their opinion toward new technologies which resulted to be as overall good (M= 4.31, SD=0.63 on a 5-point Likert scale); additionally participants reported to be quite familiar with speech-based technology (M= 3.85, SD=0.55 on a 5-point Likert scale).

### Materials and method

**Fig. 6:**
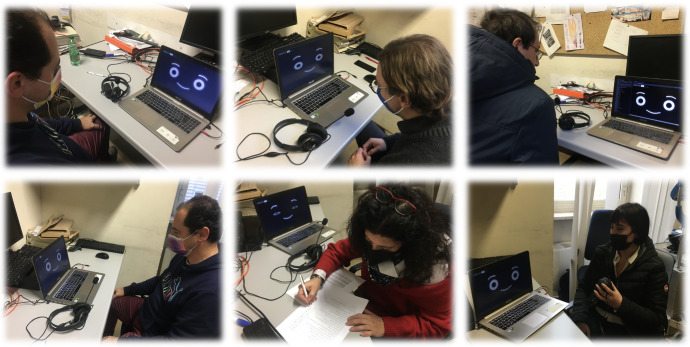
Some pictures illustrating the experimental sessions

For experimental purpose, the developed solution has been installed on a laptop with the “face” of the system visible on the screen. An external microphone has been used for the interaction. Some figures from the experimental sessions can be seen in Fig. 6. Participants were invited at the lab and were illustrated with the experimental procedure, namely that they would have filled in some questionnaires and they would have had the chance to verbally interact with a chatbot during the administration of a cognitive exercise. More in detail, the overall interaction foresaw:

1. *General information* a first part during which the system collected information about the person’s personality as to get a better knowledge about him/her and personalize the interaction. In this phase, the module of personality insight was used and tested.
2. *Cognitive excercise* the actual administration of the cognitive game articulated into two sessions (a first set of simple words, and a second set of more difficult words). During this phase, the system reacted according to the user’s personality as detected in the previous part.

After the execution of the experimental trial, participants were asked to fill in the Chatbot Usability Questionnaire (CUQ, Holmes et al. 2019) for assessing the usability. Additionally, a socio-demographic questionnaire served for collecting data about gender, age, familiarity with speech-based systems like Siri, Alexa, etc. (on a 5-point Likert scale), and opinions about new technologies (on a 5-point Likert scale). Finally, the Eysenck Personality Questionnaire (EPQ-R, Eysenck et al. 1985) was administered in this context in order to investigate the personality trait of Extroversion. More specifically, it served in order to evaluate whether the personality obtained through the system matched with those assessed through the self-report. Other metrics were collected during the experimental session: *length of the interaction*,* number of turns* (agent’s, user’s and total number), *errors* made by the agent.

### Results

Each interaction lasted on average 6.58 min (SD=1.54). During this time, the system collected the information for personalizing the dialogue according to the participant’s personality (extroverted vs introverted) and then delivered the cognitive exercise providing tailored feedback to the participant. The overall interaction consisted of an average total amount of 36.15 (SD= 6.62) turns. More in detail, the agent talked for a mean of 17.15 turns (SD= 3.28), while the user for 19 turns (SD= 3.58). The slightly higher number of turns by users was mostly due to the repetition of a same request by the participants to the agent. There was also a quite low amount of errors by the agent giving the wrong answer to a user’s question (M= 1.31, SD= 0.63). In Table 5, detailed information of the above-mentioned parameters can be seen for each participant. In general, a quite robust performance of the system emerged with a low number of errors, although only in one occasion it performed with no errors at all (Subject S02).

Participants have been also administered with a questionnaire on usability, the “Chatbot Usability questionnaire”, and the results showed positive opinion regarding the interaction with the agent. A mean score of 68.63 (SD= 11.97) out of 100 can be considered as a positive output taking into account the preliminary status of development of the system. Finally, participants’ personality have been also evaluated (EPQ) in order to investigate whether the system was able to capture this aspect through the first part of interaction. The score obtained by the system and Extroversion score from EPQ were correlated by means of Pearson statistic and a positive significant correlation emerged ( *r=* 0.68, *p=* 0.0096). This last result supports the capability of our system in capturing the user’s personality and consequently adapting its behavior accordingly.

**Table 5:** Detailed information about agent’s turns, user’s turns and errors performed during the interactions

Subject	Agent turns	User turns	Total turns	Agent errors
S01	18	17	35	2
S02	22	26	48	0
S03	23	23	46	1
S04	19	19	38	1
S05	15	16	31	1
S06	11	13	24	1
S07	16	22	38	2
S08	19	21	40	2
S09	18	20	38	1
S10	18	21	39	2
S11	15	16	31	2
S12	15	16	31	1
S13	14	17	31	1

### Limitations of this work

The involvement of real users for this preliminary testing phase led to encouraging outputs. A low number of errors occurred and the interaction was judged positively by the participants. Nevertheless some limitations need to be mentioned. First of all, a rather simple task was designed to carry out this validation session; additionally, this laboratory setting is still far from being representative of a real world setting. The runtime validation of the approach pointed out some limitations concerning the reliability of human–robot interaction. For example, the approach did not always managed overlaps between users’ and robot speeches or misunderstandings reliably. When dealing with natural language processing, misunderstanding may arise from several factors: (i) there may be background noise in the environment or the sentence pronounced by the user might be mispronounced, hence the speech to text module may fail to recognize the sentence; (ii) the sentence could be pronounced well but could have syntactic problems or it could be very different from the sentences used for the training of the intent recognition module or; (iii) the sentence is pronounced well and the intents are recognized correctly, but the user’s intention was not foreseen at a certain point in time. Another critical aspect of the current approach concerns the definition of the policy operators which, in order to define complex behaviors, easily becomes cumbersome and error prone.

## Conclusions

This paper addresses the problem of synthesizing robotic personalized assistive services and executing them obtaining flexible and adaptive dialogue-based interactions. In so doing we have integrated AI functionalities for KRR, AP and, in parallel, policy-based approaches. It is worth noting in particular that the subdivision of responsibilities between the two proposed layers allow the robot to integrate a long-term view, a sort of knowledge-based “strategist” that decides the timing of the interaction, with a dialogue-based “assistant” that allows a fine tuning of the interaction deciding contents of such an interaction.

Future works will investigate the use of reinforcement learning techniques and transformer-based models to replace the policy operators so as to simplify the work entrusted to the expert user to enhance the adaptability and reliability of the interactions. Additionally, future work is planned to involve older users in an ecological environment for a long-term investigation and to consider additional cognitive exercises and assistive services (e.g., reminders and health monitoring). This will allow us gathering more robust evidences in terms of both personalization capabilities and effectiveness of the approach as a support for personalised daily assistance.

## Footnotes

1 The present work is a significantly extended version of De Benedictis et al. (2020).

2 https://www.who.int/standards/classifications.

3 https://jena.apache.org/index.html.

4 https://protege.stanford.edu.

5 https://bioportal.bioontology.org/ontologies/ICF.

6 The reactive reasoner uses the NLU module of the IBM’s Watson Assistant service - https://www.ibm.com/cloud/watson-assistant.

7 We use IBM’s Watson Speech to Text service: https://www.ibm.com/cloud/watson-speech-to-text.

8 The reactive reasoner uses the IBM’s Watson Personality Insight service: https://www.ibm.com/cloud/watson-personality-insights.

9 The reactive reasoner uses the IBM’s Watson Natural Language Understanding service: https://www.ibm.com/cloud/watson-natural-language-understanding.
